# Osteocalcin Ameliorates Motor Dysfunction in a 6-Hydroxydopamine-Induced Parkinson’s Disease Rat Model Through AKT/GSK3β Signaling

**DOI:** 10.3389/fnmol.2018.00343

**Published:** 2018-09-27

**Authors:** Xing-zhi Guo, Chang Shan, Yan-fang Hou, Geng Zhu, Bei Tao, Li-hao Sun, Hong-yan Zhao, Guang Ning, Sheng-tian Li, Jian-min Liu

**Affiliations:** ^1^Department of Endocrine and Metabolic Diseases, Shanghai Clinical Center for Endocrine and Metabolic Diseases, Rujin Hospital, Shanghai Jiao Tong University School of Medicine, Shanghai Institute of Endocrine and Metabolic Diseases, Shanghai, China; ^2^Bio-X Institutes, Key laboratory for the Genetics of Development and Neuropsychiatric Disorders (Ministry of Education), Shanghai Key Laboratory of Psychotic Disorders, and Brain Science and Technology Research Center, Shanghai Jiao Tong University, Shanghai, China

**Keywords:** osteocalcin, Parkinson’s disease, 6-hydroxydopamine, astrocyte, microglia, AKT/GSK3β

## Abstract

Osteoblasts derived osteocalcin (OCN) is recently reported to be involved in dopaminergic neuronal development. As dopaminergic neuronal injury in the substantia nigra (SN) is a pathological hallmark of Parkinson’s disease (PD), we investigated whether OCN could exert protective effects on 6-hydroxydopamine (6-OHDA)-induced PD rat model. Our data showed that the OCN level in the cerebrospinal fluid (CSF) in PD rat models was significantly lower than that in controls. Intervention with OCN could improve the behavioral dysfunction in PD rat models and reduce the tyrosine hydroxylase (TH) loss in the nigrostriatal system. In addition, OCN could inhibit the astrocyte and microglia proliferation in the SN of PD rats. *In vitro* studies showed that OCN significantly ameliorated the neurotoxicity of 6-OHDA through the AKT/GSK3β signaling pathway. In summary, OCN plays a protective role against parkinsonian neurodegeneration in the PD rat model, suggesting a potential therapeutic use of OCN in PD.

## Introduction

Parkinson’s disease (PD) is one of the most common neurodegenerative diseases characterized by cardinal movement disorders, including bradykinesia, tremor and rigidity (Postuma et al., 2015; Obeso et al., 2017), and its nonmotor symptoms, such as cognitive dysfunction, are also increasingly recognized (Crowley et al., 2018). Dopaminergic neuronal degeneration, with the consequences of loss of dopaminergic neurons in the substantia nigra (SN) and depletion of dopamine (DA) in the striatum, are the main pathological events in PD (Maetzler and Berg, 2018).

Since its first description 200 years ago, numerous efforts have been made to explore the underlying mechanisms of PD (Balestrino and Schapira, 2018) and to identify molecules inside (Parisiadou et al., 2014; Steinkellner et al., 2018) and outside of the brain (Weng et al., 2007; Chou et al., 2008; Guo et al., 2017) responsible for disease development or progression. Due to its complexity and heterogeneity in pathogenesis, there is also a need to think about PD in new ways and find novel means to manage this debilitating disorder (Maetzler and Berg, 2018). Increasing clinical evidence indicated that patients with PD have a higher susceptibility to another very common age-related disease–osteoporosis (Torsney et al., 2014). The cross-talk between bone and brain was suggested by a series of works from Karsenty’s group revealing that bone-derived osteocalcin (OCN) is important for brain development (Obri et al., 2018).

OCN a 49-amino acid peptide highly conserved among species, is exclusively synthesized in bone by osteoblasts and thus was traditionally regarded as a bone formation marker. However, several extraskeletal physiological functions of OCN are continually revealed, including modulation of energy metabolism (Wei et al., 2015; Liu et al., 2016), muscle power (Mera et al., 2016a,b) and male fertility (Karsenty and Oury, 2014) through its receptor GPRC6A in the peripheral tissues. More importantly, OCN passes through the blood brain barrier (BBB) and binds to neurons in the midbrain, brainstem and hippocampus. In the midbrain, OCN binds to neurons in the ventral tegmental area (VTA), which is enriched in dopaminergic neurons, and facilitates the synthesis of DA. OCN could also enhance the synthesis of other monoamine neurotransmitters, such as serotonin and norepinephrine, while inhibiting GABA production by regulating the expression of the key enzymes implicated in the synthesis of these neurotransmitters, with the consequence of preventing anxiety and favoring spatial learning and memory in mice (Oury et al., 2013). All these central effects of OCN are accomplished through its newly identified second receptor, GRP158, in the hippocampus (Khrimian et al., 2017b).

Thus, though the molecular mechanism responsible for the degeneration of dopaminergic neurons remains elusive, this progress in revealing the novel role of OCN for brain development and age-related cognitive decline prompted us to hypothesize and test whether OCN could exert protective effects in PD. The rational for such a study is as follows: (1) OCN could bind to the midbrain neurons and increase the levels of DA neurotransmitters (Oury et al., 2013), which are significantly decreased in the midbrain of PD patients (Paul et al., 2018); (2) mice with defective bone formation demonstrate increased anxiety-like behavior, impaired spatial learning and memory due to its reduced circulating OCN levels and diminished expression of OCN target genes in the brain (Khrimian et al., 2017a); and (3) with aging, serum OCN concentration also decreases in mice (Khrimian et al., 2017b) and humans (Liu et al., 2008).

To test the novel hypothesis that OCN exerts protective effects in PD, in the current study, we investigated whether intrastriatal (i.s.) injection or intraperitoneal (i.p.) injection of OCN could ameliorate the behavioral deficits in 6-hydroxydopamine (6-OHDA)-induced PD rat models and whether OCN treatment could reduce dopaminergic neuronal loss in the nigrostriatal pathway. Since AKT/GSK3β signal transduction impairment is believed to be associated with dopaminergic neuronal dysfunction in PD (Morissette et al., 2010; Chen et al., 2017), we also explored whether the protective function of OCN on SN dopaminergic neurons is mediated via AKT/GSK3β signaling.

## Materials and Methods

### Animals and Surgical Procedure

Sprague-Dawley (SD) male rats (250–290 g) and C57BL/6 male mice (25–30 g) were provided by SLACCAS Laboratory Animal Co., Ltd. (Shanghai, China). All animal experiments were performed in accordance with the National Institutes of Health Guidelines for the Care and Use of Laboratory Animals (NIH Publications No. 8023, revised 1978). The protocol was approved by the Animal Ethics Committee of Shanghai Jiaotong University. Animals were housed with free access to food and water and maintained under a 12-h light/dark cycle.

In the OCN i.p. injection experiments, 40 rats were divided into four groups: sham group, 6-OHDA-lesioned group and 6-OHDA+OCN (4 μg/kg or 40 μg/kg) groups. Four rats were dead before the behavioral test. 6-OHDA was dissolved in saline containing 0.02% (w/v) ascorbic acid to prevent auto-oxidation of 6-OHDA. To achieve unilateral lesions of the nigrostriatal system, 32 μg (4 μg/μl in 8 μl) of 6-OHDA was injected into the right medial forebrain bundle (MFB) according to a previously described procedure (Boix et al., 2015). The stereotaxic injection was based on the rat brain in stereotaxic coordinates of Paxinos and Watson (Paxinos and Franklin, 2004). Briefly, rats were anesthetized with 1% pentobarbital sodium (0.8 ml/kg) and were placed in a stereotaxic device. After the scalp was incised at the midline, two holes were drilled through the skull on the right side according to the two following coordinates (a, AP: 3.7 mm, ML: 1.7 mm, and DV: 7.8 mm; b, AP: 4.4 mm, ML: 1.2 mm, and DV: 7.8 mm). To prevent 6-OHDA-induced damage to noradrenergic neurons, the rats were given desipramine (25 mg/kg) by an i.p. injection 30 min before the delivery of 6-OHDA. A single injection of 6-OHDA was performed at the above two holes using a 10-μl Hamilton syringe at a rate of 4 μl/4 min. After injection, the needle was left in the brain for another 10 min and was then withdrawn slowly. To evaluate the protective effect of OCN on the PD rat model, 4 μg/kg or 40 μg/kg OCN was intraperitoneally injected once a day for 2 days before 6-OHDA injection and lasted for 1 week.

In the OCN i.s. injection experiments, 66 rats were randomly divided into six groups: sham group, 6-OHDA-lesioned group, sham+OCN (4 μg) group, 6-OHDA+OCN (0.4 μg) group, 6-OHDA+OCN (4 μg) group and 6-OHDA+nerve growth factor (NGF, 1 μg) group. Since NGF was reported to protect against 6-OHDA-induced PD rat model and increase dopaminergic neuronal survival, the NGF was used as a positive control in this study (Salinas et al., 2003; Chaturvedi et al., 2006). Six rats were dead before the behavioral test. To achieve unilateral lesions of the nigrostriatal system, 20 μg (5 μg/μl in 4 μl) of 6-OHDA was injected into the right striatum according to a previously described procedure (Lindholm et al., 2007). One-time injections of 6-OHDA, OCN (0.4 or 4 μg) or saline were performed using a 10-μl Hamilton syringe at a rate of 4 μl/4 min. After injection, the needle was left in the brain for another 10 min and was then withdrawn slowly. OCN was injected into the striatum at the same coordinates (AP: 0.7 mm; ML: 3 mm; and DV: 5 mm) 4 h before the injection of 6-OHDA. The doses of 6-OHDA, desipramine and OCN used in the experiment were determined according to preliminary experiments. The schedule of the experiment is presented in Supplementary Figure S1.

Limited by the species reactivity of the OCN, GPR158 and GPRC6A antibodies, coimmunoprecipitation was performed on mouse striatum. Three mice were used for coimmunoprecipitation each time, and the assay was repeated four times, for a total of 12 mice used in the coimmunoprecipitation assay.

### Open-Field Test

The open-field test (OFT) was used to evaluate locomotor activity changes in the 6-OHDA PD rat model as described previously (Walsh and Cummins, 1976). The OFT apparatus comprises a black rectangular box (90 × 90 × 50 cm) with an open top. The test was performed by placing each rat in the center of the box with low light (30 lux) and allowing the rat to explore for 10 min. All locomotor activity was recorded by a video camera over the box. The movement distance and rearing frequency were calculated with the ANY-maze automated video tracking system to reflect the movement and exploration activity of the rats (Version 4.115; Stoelting Co., Wood Dale, IL, USA). The rearing frequency were recorded by an investigator who was blind to the treatment group.

### Locomotor Asymmetry Tests

The cylinder test and the elevated body swing test (EBST) were used to evaluate the locomotor asymmetry of rats after unilateral (right) 6-OHDA injection-induced dopaminergic neuronal loss (Borlongan and Sanberg, 1995). Both tests were performed at 6 weeks postlesion during the onset of the dark period when rats are more active. The behavioral results were scored by assessor who were blind to the original treatment classification.

For the cylinder test, the use of each forelimb during exploratory activity was calculated as previously described (Vercammen et al., 2006). Briefly, each rat was placed in a clear cylinder (40 cm diameter, 60 cm height) for 3 min without any habituation prior to recording. The use of the ipsilateral forelimb, contralateral forelimb, or both forelimbs during rat rearing was recorded by a blinded examiner. The results are presented in terms of the ratio of contralateral forelimb (left) wall touches relative to the number of touches by ipsilateral forelimb (right). Non-lesioned control rats should score approximately 1 in this test, while this ratio is supposed to decrease in unilateral 6-OHDA-lesioned rats.

The EBST was developed by (Borlongan and Sanberg, 1995) to evaluate movement impairment in rats with unilateral nigrostriatal DA depletion (hemiparkinsonism), which exhibited significantly biased swings toward the contralateral side (Borlongan and Sanberg, 1995). In the EBST, each rat was placed in a neutral position and was held 1–2 cm from the base of its tail. Then, the rats were elevated vertically to 1 cm above the surface for 1 min. A swing was counted whenever the rat moved its head 30° from the vertical axis to either side. To record an additional swing, the rat had to first return to the vertical axis. If the rat stopped swinging in the vertical position for more than 5 s, the tail was gently pinched to induce swinging. The test was conducted by two blinded observers, with one responsible for holding the rat and the other for recording and timing the session. The total number of swings to each side was quantified, and the contralateral swings (left) were divided by the number of ipsilateral swings to get the percentage of left swings. Right striatum or MFB 6-OHDA-lesioned rats exhibited significantly biased swing activity toward the left side.

### Immunohistochemistry and Cell Counting

Six weeks after the 6-OHDA-injection, rats were deeply anesthetized with 1% pentobarbital sodium (0.8 ml/kg), perfused transcardially with 0.9% saline and fixed with 4% paraformaldehyde (PFA). Brains were dissected and post fixed for an additional 24 h in 4% PFA and were then sequentially immersed into 20% sucrose and 30% sucrose solutions. Coronal sections of 30-μm thickness were prepared using a freezing sliding microtome. Endogenous peroxidase activity was inactivated with 0.3% H_2_O_2_ for 30 min. The sections were permeabilized with 0.3% Triton X-100, blocked with 5% goat serum, and incubated with primary antibodies against TH overnight at 4°C. The sections were washed three times for at least 30 min and were incubated for 2 h at room temperature with a biotin-conjugated goat anti-rabbit IgG secondary antibody. The sections were washed three times for at least 30 min and incubated with HRP-conjugated avidin for 1 h at room temperature. The sections were rinsed three times for at least 30 min and then incubated with diaminobenzidine (DAB) for 3 min. Images were obtained using a microscope (Olympus IX70, Japan). The number of TH-positive neurons in the SN was counted with the optical dissector method in Image-Pro Plus software 6.0 (Media Cybernetics) as previously described. Every fifth section was quantified, and data are expressed as a percentage of TH^+^ neurons on the impaired side relative to the intact side.

### Immunofluorescence and Cell Counting

The process to prepare coronal sections was the same as that used for immunohistochemistry. Brains were dissected and postfixed for an additional 24 h in 4% PFA and were then sequentially immersed into 20% sucrose and 30% sucrose solutions. Coronal sections of 30-μm thickness were prepared using a freezing sliding microtome. The sections were permeabilized with 0.3% Triton X-100, blocked with 5% goat serum, and incubated with primary antibodies against TH and Iba1 or GFAP and Nestin overnight at 4°C. The sections were washed three times for at least 30 min and were incubated for 2 h at room temperature in the dark with a FITC- or PE-conjugated goat anti-rabbit or mouse IgG secondary antibody and DAPI. The sections were washed three times for at least 30 min in the dark. Images were obtained using a fluorescence microscope (AMG EVOS FL Microscope, USA). Every fifth section was quantified, and data are expressed as a percentage of GFAP- or Iba1-positive neurons on the impaired side relative to the intact side.

### Cell Culture and Viability

PC12 cells, a line of cultured rat pheochromocytoma cells that have been established for decades as a particularly useful cell model for studying PD (McGuire et al., 1978), were cultured in Dulbecco’s Modified Eagle’s Medium (DMEM) supplemented with 10% horse serum, 5% fetal bovine serum (FBS) and 1% penicillin and streptomycin. The cells were maintained in a 37°C incubator with 5% CO_2_, and the medium was refreshed every 2–3 days.

Cell Counting Kit-8 (CCK-8), which allows for the determination of cell viability in cell proliferation and cytotoxicity assays by utilizing Dojindo’s highly water-soluble tetrazolium salt, was used to detect cell viability according to the manufacturer’s protocol as previously described (Shoji et al., 2009).

Briefly, PC12 cells were seeded in 96-well plates at a density of 5 × 10^3^ cells per well. OCN (100 ng/ml) was administered 2 h before the addition of 6-OHDA (100 μM), and the AKT phosphorylation inhibitor LY294002 (20 μM) was administered 30 min before the addition of OCN. After PC12 cells were treated for 24 h as described above, CCK-8 (10 μl) solution was added to each well, and the cells were incubated for 1 h. The highly water-soluble tetrazolium salt (WST-8) in the CCK-8 kit was reduced to a yellow-colored formazan dye by the dehydrogenase activity in the cells. The amount of the soluble formazan dye in the cell culture media was directly proportional to the number of living cells. Finally, the optical absorbance was detected at 450 nm using a microplate reader. High optical density indicated high cell viability, and the presented data were normalized to the control data.

All *in vitro* assays in this study were conducted three times independently, and the data were collected to calculate the overall effects. To evaluate the direct effect of OCN on 6-OHDA, we performed an assay to monitor the formation of p-quinone and thiol conjugate, which resulted from the autoxidation of 6-OHDA, through detecting the optical absorbance at 490 nm for p-quinone or at 350 nm for thiol conjugate as previously described.

### Flow-Cytometric Analysis

PE-Annexin V (AV) and 7-amino-actinomycin (7-AAD) were used to quantify cell apoptosis induced by 6-OHDA according to the manufacturer’s protocol. Briefly, after cells were treated as described, the cells were detached from the dishes and washed twice with cold PBS. Then, the cells were resuspended in binding buffer at a concentration of 1 × 10^6^ cells/ml. A total of 100 μl of solution (containing 1 × 10^5^ cells) was transferred to a 5 ml culture tube and incubated with 5 μl of PE-AV and 5 μl of 7-AAD for 15 min at room temperature in the dark. After 400 μl of binding buffer was added to each tube, the cells were analyzed by flow cytometry within 1 h. The number of apoptotic cells was counted in the R2 (AV^+^/7-AAD^+^, the late phase apoptotic cells) and R4 (AV^+^/7-AAD^−^, the early phase apoptotic cells) regions.

### Western Blotting

Cells or brain tissues were lysed in RIPA lysis buffer to extract total cellular protein. The protein concentration was measured by BCA protein assay reagents, and equal amounts of proteins were subjected to 10% or 15% SDS-PAGE according to protein molecular weight. Electrophoresed proteins were then transferred to polyvinylidene fluoride membranes and blocked with 5% (w/v) nonfat dry milk in Tris-buffered saline at room temperature before an overnight incubation at 4°C with various primary antibodies. After extensive washing, the appropriate HRP-conjugated secondary antibody was diluted (1:5,000) in blocking milk containing 0.1% Tween 20 and incubated for 2 h. Blots were developed with an ECL reagent. Integrated optical density measurement and analysis were conducted with Gel-Pro Analyzer software. The primary antibodies used were as follows: anti-bcl-2 (1:1,000), anti-caspase-3 (1:1,000), anti-p-AKT (1:1,000), anti-AKT (1:1,000), anti-p-GSK3β (1:1,000), anti-GSK3β (1:1,000) and anti-GAPDH (1:5,000). GAPDH was used as an internal control.

### Quantitative PCR for GPR158 and GPRC6A Expression in Rat Brain

Quantitative polymerase chain reaction (q-PCR) was conducted to determine the expression of GPR158 mRNA in various brain tissues from 8-week-old SD rats. RNA from brain samples of rats was extracted with TRIzol reagent. RNA quality was evaluated with a Nano Bioanalyzer, and reverse transcribed into cDNA using a GoScript™ Reverse Transcription System. The cDNA was used in PCR amplification with the following primers for gpr158 (NM_001170326.1; forward: 5′-CTGCTCGCTCATCTGGGATTG-3′ and reverse: 5′-TCCAGGGAATAGAGGGGTCTG-3′), gprc6a (NM_001271106.1; forward: 5′-ATCCGCTTTCCTTCGTTT-3′ and reverse: 5′-ATCGTCGGTTGTTATGGC-3′) and gapdh (NM_017008.4; forward: 5′-CAGCCGCATCTTCTTGTGC-3′ and reverse: 5′-ATCCGTTCACACCGACCTTC-3′).

### Coimmunoprecipitation

To determine the roles of GRP158 and GPRC6A in OCN function in mouse striatum, striata were dissected on ice and homogenized in NP-40 buffer with 1× protease and phosphatase inhibitor cocktail. Homogenized striata were centrifuged at 12,000× *g* for 20 min at 4°C. The supernatants were preset with 30 μl of protein G agarose beads and then incubated for 2 h at 4°C with anti-OCN antibody coupled to 30 μl of protein G agarose beads followed by three NP-40 washes. Immunocomplexes were resolved in 2× SDS-PAGE, heated at 100°C for 5 min and then analyzed by western blotting as described above.

### CSF Extraction and Hormonal Measurement

Collection of cerebrospinal fluid (CSF) from the cisterna magna in rats was performed according to a previously described protocol with some modification (Liu and Duff, 2008). Briefly, the rats were anesthetized by 1% pentobarbital sodium (0.8 ml/kg) and then were placed in astereotaxic device. The skin of the neck was shaved, and the surgical site was swabbed with 10% povidone iodine, followed by 70% ethanol. A sagittal incision of the skin was made inferior to the occiput, and the subcutaneous tissue and muscles were separated under the dissection microscope. After the CSF space was visible, a 1-ml syringe was inserted into the cisterna magna, and then the CSF was extracted.

Serum and CSF levels of OCN were determined with a rat OCN ELISA kit according to the manufacturer’s protocol. Briefly, samples were diluted prior to the assay and 25 μl of standard or diluted samples were pipetted into the designated wells. Afterwards, 50 μl of biotinylated rat OCN antibody was pipetted into the wells, and the plate was incubated at room temperature for 1 h on a horizontal rotator at 180–220 RPM with one plate sealer. Wells were then washed five times, and 100 μl of HRP-conjugated rat OCN antibody were added into each well next. The plate was incubated at room temperature for another 1 h as previously described, followed by five washes, and then, 100 μl of ELISA HRP substrate was added. After a 30-min incubation, 50 μl of ELISA stop solution was pipetted into each well and mixed for 1 min. The absorbance was read at 450 nm with a microtiter plate reader within 10 min.

### Materials

6-OHDA hydrobromide, desipramine hydrochloride, LY-294002 hydrochloride, TRIzol reagent, DAPI and Hoechst-33258 were obtained from Sigma (St. Louis, MO, USA). DMEM and heat-inactivated endotoxin-free FBS were purchased from Gibco (Logan, UT, USA). AGoScript™ Reverse Transcription System was obtained from Promega (Madison, WI, USA). Protein extraction kits were obtained from Beyotime Biotechnology (Wuhan, China).

Recombinant OCN was from Bachem (Bubendorf, Switzerland, Catalog: H-6552). A rat OCN ELISA kit was purchased from MyBioSource (San Diego, CA, USA, Catalog: MBS2022619). An OCN antibody was obtained from Santa Cruz Biotechnology (Santa Cruz, CA, USA). Cell viability was evaluated with a Cell Counting Kit-8 (CCK-8) assay (Dojindo Molecular Technologies, Inc., Rockville, MD, USA). An antibody against tyrosine hydroxylase (TH) was from Millipore (Cambridge, MA, USA). Antibodies against GFAP, Iba1, Nestin, bcl-2, caspase-3, p-AKT, AKT, p-GSK3β, GSK3β and GAPDH, as well as all secondary antibodies, were obtained from Cell Signaling Technology (Beverly, MA, USA). A PE AV Apoptosis Detection Kit I was purchased from BD PharMingen™ (San Diego, CA, USA, Catalog: 559763), and an ABC kit was obtained from Vector Laboratories, Inc. (Burlingame, CA, USA). The phosphatase inhibitor cocktail was from Thermo Fisher Scientific (Fair Lawn, NJ, USA), and the protein G agarose beads were from GE Healthcare (Piscataway, NJ, USA).

### Statistical Analysis

The data were analyzed by one-way analysis of variance (ANOVA) followed by *post hoc* comparison using Bonferroni’s test with SPSS 20.0 (IBM, Armonk, NY, USA). All data were normally distributed. Data are expressed as the mean ± SEM. *P* < 0.05 was considered statistically significant.

## Results

### The OCN Levels in Serum and CSF and Its Receptors in the Brain

**Figure 1:**
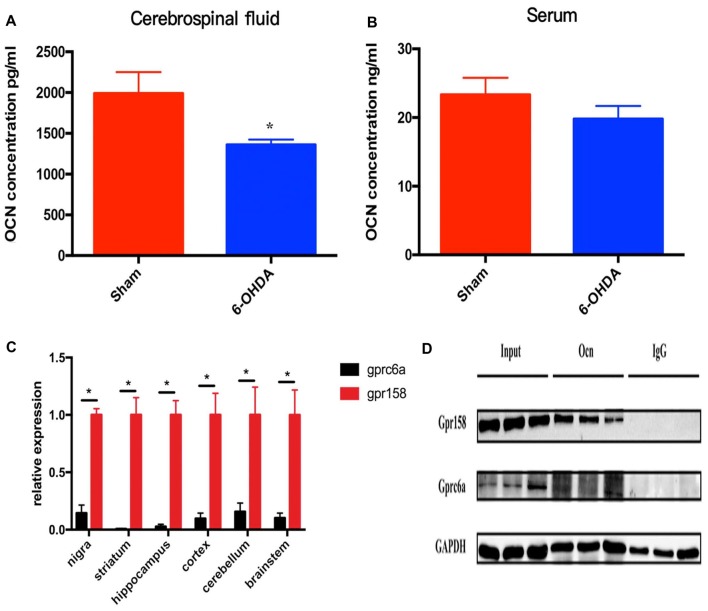
The levels of osteocalcin (OCN) in the serum or cerebrospinal fluid (CSF) of control and 6-hydroxydopamine (6-OHDA)-induced Parkinson’s disease (PD) rat models. The CSF OCN levels in PD rats dramatically decreased compared to control rats (**A**, *n* = 17). No difference in the serum OCN levels was found between control and PD rats (**B**, *n* = 19). Expression of GPR158 and GPRC6A in various brain-derived tissues of 8-week-old rats (**C**, *n* = 3). Coimmunoprecipitation of 8-week-old mice striata and proteins were then subjected to western blot analysis using anti-Gpr158 and anti-GPRC6a antibodies (**D**, *n* = 3). Values were obtained from three independent experiments and results are given as the mean ± SEM. *P < 0.05.

In this study, we first measured the serum and CSF levels of OCN in these rats. It was shown that the CSF level of OCN was significantly lower in PD rat models than that in controls (*P* = 0.026, Figure 1A). The serum OCN concentration in PD rat models and controls were comparable (*P* = 0.319, Figure 1B).

GPRC6A and GPR158 are the only two known receptors of OCN in mice. The q-PCR results displayed a much higher mRNA expression level of GPR158 than that of GPRC6A in all the rat brain areas tested, with the relative expression reaching 6.9 times higher in the SN (*P* = 0.020), 159.9 times higher in the striatum (*P* = 0.023), 37.9 times higher in the hippocampus (*P* = 0.018), 10.3 times higher in the cortex (*P* = 0.040), 6.4 times higher in the cerebellum (*P* = 0.046) and 9.8 times higher in the brainstem (*P* = 0.036), respectively (Figure 1C). In addition, the coimmunoprecipitation results demonstrated OCN bound GPR158 in the striatum, while the amount of GPRC6A bound with OCN was drastically lower than GPR158 (Figure 1D).

### OCN Treatment Improves Behavioral Deficits in PD Rats

**Figure 2:**
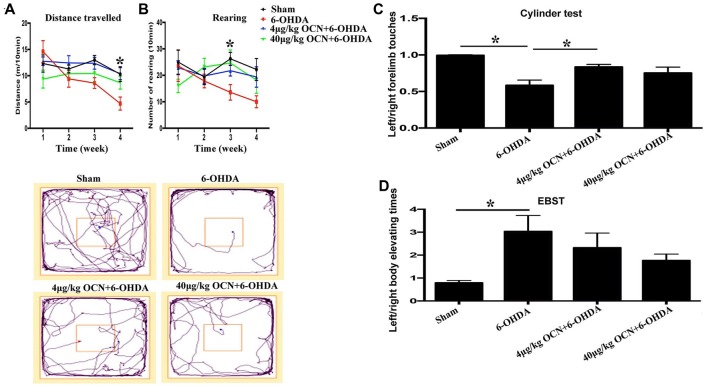
Effect of intraperitoneal (i.p.) injection of OCN on distance traveled **(A)** and rearing responses **(B)** in the open-field test (OFT). The cylinder test **(C)** and elevated body swing test (EBST; **D**) were used to evaluate locomotor asymmetry. Compared with the sham groups, 6-OHDA injection decreased the distance traveled by rats in the OFT, and reduced the use of contralateral (left) forelimb in the cylinder test. The injection of 4 μg/kg OCN significantly improved the movement distance of 6-OHDA-lesioned rats in the 4th week (*P* < 0.001), while 40 μg/kg OCN increased the number of rearing responses in the 3rd week (*P* < 0.05). The representative traces of the OFT are shown in the lower panel of **(A,B)**. In the cylinder test, 4 μg/kg OCN improved the use of the contralateral forelimb (*P* < 0.05). No obvious differences were observed in the EBST. *n* = 8–10, *P < 0.05 (compared with the 6-OHDA group).

Rats that received i.p. injection of OCN were subjected to the OFT first. It was revealed that within the group, the movement distance in 6-OHDA-injected rats were reduced over time with significant differences seen in the 3rd week (8.59 ± 0.88 m vs. 14.57 ± 1.79 m, *P* < 0.001) and 4th week (4.71 ± 0.97 m vs. 14.57 ± 1.79 m, *P* < 0.001) compared with the 1st week, while such changes were not observed in 6-OHDA-injected rats receiving 4 μg/kg or 40 μg/kg OCN over the 4 weeks (Figure 2A, Supplementary Table S1). When comparing between the groups, the movement deficit in 6-OHDA-injected rats was apparent in the 3rd (*P* < 0.05) and 4th weeks (*P* < 0.001) compared with sham rats. OCN treatment at a dosage of 4 μg/kg markedly increased the movement distance in 6-OHDA rats in the 4th week (10.37 ± 1.01 m vs. 4.71 ± 0.97 m, *P* < 0.001; Figure 2A, Supplementary Table S1).

Rearing is a parameter that reflects rat forelimb movement activity and exploration. As shown in Figure 2B, the frequency of rearing in the 6-OHDA-lesioned group declined with time over 4 weeks (4th week, 10.00 ± 1.94 vs. 1st week, 23.13 ± 7.73, *P* < 0.05). However, this movement dysfunction was not observed in 6-OHDA-lesioned drats receiving either 4 μg/kg or 40 μg/kg OCN i.p. treatments. Further analysis showed that the number of rearing in the 40 μg/kg OCN treatment group was significantly increased compared to those in the 6-OHDA group in the 3rd week (24.78 ± 4.49 vs. 13.63 ± 2.57, *P* < 0.05). The representative traces of OFT are shown in the lower panel of Figures 2A,B.

In the cylinder test, the frequency of left forelimb touches was markedly reduced in PD rat models compared to the sham group (0.585 ± 0.062 vs. 0.997 ± 0.015, *P* < 0.001), but this deficit was reversed by OCN intervention, with significant improvement seen in the 4 μg/kg OCN group (0.835 ± 0.031 vs. 0.585 ± 0.062, *P* < 0.05, Figure 2C) but not in the 40 μg/kg group. The results from the EBST revealed that the left/right elevating times in 6-OHDA rats were significantly higher than those in the sham group (3.031 ± 0.649 vs. 0.797 ± 0.077, *P* < 0.05), and with the treatment of OCN (both 4 μg/kg and 40 μg/kg), this abnormality tended to improve but was not significantly different compared with the 6-OHDA rats (Figure 2D).

**Figure 3:**
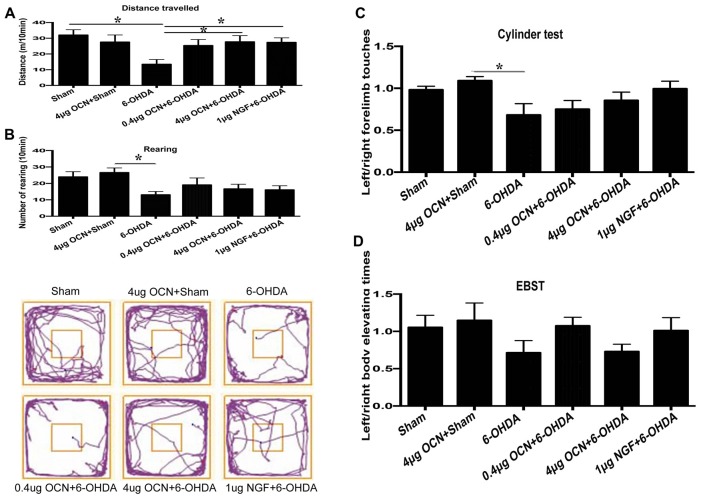
Effect of intra-striatum injection of OCN on distance traveled **(A)**, rearing responses **(B)** in OFT. The cylinder test **(C)** and EBST **(D)** were used to evaluate locomotor asymmetry. Compared with the sham groups, 6-OHDA injection decreased the distance traveled by rats, while the injection of OCN (4 μg) or nerve growth factor (NGF) significantly improved movement distance **(A)**, but not rearing impairment **(B)** of 6-OHDA-lesioned rats in OFT. The representative traces of the OFT are shown in the lower panel of **(B)**. No obvious differences were observed in the cylinder test **(C)** or the EBST **(D)**. *n* = 9–11, *P < 0.05 (compared with the 6-OHDA group).

To further investigate whether OCN has a direct central action in PD rat models, OCN and 6-OHDA were injected directly into the striatum (i.s.), and rat movement dysfunctions were examined 6 weeks later. We found that the movement distance in the 6-OHDA-injected group was less than that of the sham group (13.53 ± 2.67 m vs. 31.92 ± 3.08 m, *P* < 0.001), which was significantly ameliorated by either OCN (4 μg, i.s.; 27.80 ± 3.88 m vs. 13.53 ± 2.67 m, *P* < 0.05) or NGF (1 μg, i.s.; 27.43 ± 2.49 m vs. 13.53 ± 2.67 m, *P* < 0.05) intervention (Figure 3A, Supplementary Table S2).

The frequency of rearing in the 6-OHDA-lesioned group declined compared to that in the sham group (13.11 ± 1.78 vs. 24.00 ± 2.72, *P* < 0.05), both OCN and NGF seemed to alleviate the forelimb movement dysfunction induced by 6-OHDA, but all the changes were not significant (Figure 3B). The representative traces of OFT are shown in the lower panel of Figure 3B.

In the cylinder test, there was a reduction (~25%) in contralateral forelimb use in the 6-OHDA-lesioned group compared to that of the sham group, but it was not significant. OCN i.s. treatments led to a trend of improvement but without significant differences (Figure 3C).

The EBST results indicated that there was a slight decline in contralateral swings (toward the left side) in the 6-OHDA-lesioned group compared to those in the sham group, while there were no significant differences in the EBST among all the included groups (Figure 3D).

### OCN Treatment Reduces Dopaminergic Neuronal Loss in the Nigrostriatal Pathway

**Figure 4:**
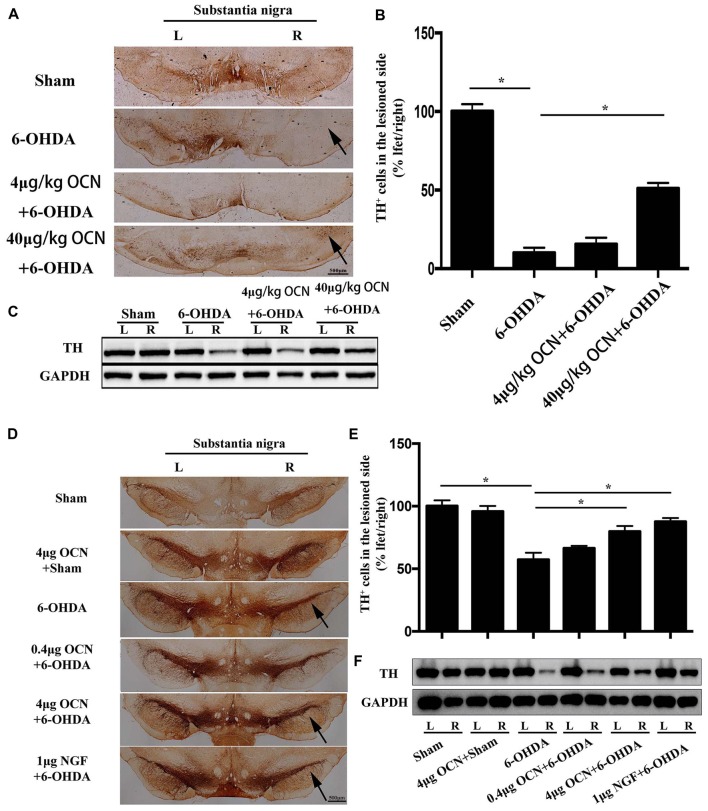
The protective effect of OCN on tyrosine hydroxylase (TH^+^) neurons and fibers in the nigrostriatal system in the unilateral 6-OHDA-lesioned PD rat model. 6-OHDA induced ~90% dopaminergic neuronal loss on the lesioned side compared to the intact side of the substantia nigra (SN), which was improved by i.p. OCN injection **(A)**. Similarly, the decrease in TH-positive cell number in the SN of the lesioned side was also significantly alleviated by OCN injection **(B)**. The results from western blots measuring TH were consistent with immunohistochemistry in the striatum **(C)**. In the intra-striatum injection OCN group, 6-OHDA induced ~50% dopaminergic neuronal loss in the lesioned side compared to the intact side of the SN, which was improved by OCN injection **(D)**. Similarly, the decrease in TH-positive cell number in the SN of lesioned side was also significantly alleviated by intra-striatum OCN injection **(E)**. The results from western blots measuring TH were consistent with immunohistochemistry in the striatum **(F)**. *n* = 16, *P < 0.05 (compared with the 6-OHDA group).

The results from TH immunostaining in the SN showed that nearly 90% of the dopaminergic neuronal loss was observed on the lesioned side (right) compared to the intact side (left) in the 6-OHDA-lesioned rats (*p* < 0.001), whereas i.p. injection of 40 μg/kg OCN significantly reversed the TH-positive neuron loss (~40% of the intact side; *P* < 0.001, Figures 4A,B).

Since the striatum tissue is the projected position for dopaminergic fibers from the SN, we next measured the TH levels in the striatum through western blot analysis. The results showed that TH expression in the striatum decreased in the 6-OHDA-lesioned group and that TH expression was markedly improved when OCN was given intraperitoneally at 40 μg/kg (*P* < 0.001, Figure 4C).

As displayed in Figures 4D,E, injection of 6-OHDA directly into the striatum resulted in nearly 50% dopaminergic neuronal loss on the lesioned side (right) compared to the intact side (left), whereas 4 μg OCN i.s. significantly reversed the TH-positive neuron loss (~80% of the intact side; *P* < 0.001, Figures 4D,E). A similar finding was also observed in the NGF group (*P* < 0.001, Figure 4D). The results from the western blot analysis also showed that the 6-OHDA-induced lower expression of TH in the striatum was markedly improved in the OCN (*P* < 0.001) or NGF (*P* < 0.001) groups (Figure 4F).

### OCN Treatment Inhibits Inflammation and Reduces the Proliferation of Microglia and Astrocytes in the SN and Striatum

**Figure 5:**
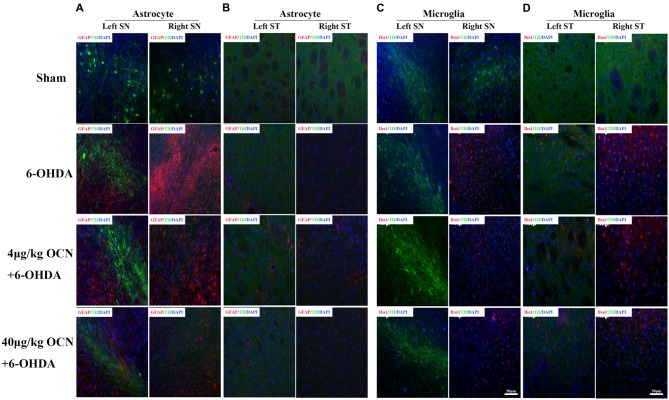
The effect of OCN on the proliferation of astrocytes and microglia. 6-OHDA significantly increased the number of GFAP-positive astrocytes in the lesioned side compared to the intact side both in the SN **(A)** and striatum **(B)**, which was inhibited by OCN injection. The same function of OCN on microglial proliferation was also observed, which was stained with Iba1 **(C,D)**. *n* = 16. Green = TH, Red = GFAP or Iba1, Blue = Nucleus.

Overexpression of the pro-inflammatory factors such as TNF-α and IL-1β is a common feature in PD (Alam et al., 2016), we measured the mRNA levels of TNF-α and IL-1β in the striatum. It was found that OCN given systemically had a tendency to reduce the expression of TNF-α and IL-1β in the striatum (Supplementary Figure S2). Overactivated microglia and astrocytes were associated with the chronic inflammatory reaction in PD (Booth et al., 2017; Liddelow et al., 2017). Thus, we stained GFAP (astrocyte)- and Iba1 (microglia)-positive glia in the SN and striatum. In the SN, a dramatic proliferation of astrocytes on the lesioned side (right) compared to the intact side (left) in the 6-OHDA-lesioned rats was observed, and this phenomenon was significantly inhibited by OCN treatments (both 4 μg/kg and 40 μg/kg; *P* < 0.001, Figure 5A). Similar findings were also observed in the striatum area (*P* < 0.05, Figure 5B). The results from the Iba1 staining experiment showed that OCN significantly inhibited the upregulation of Iba1-positive microglia numbers in the SN (*P* < 0.05) and striatum (*P* < 0.05) stimulated by 6-OHDA (Figures 5C,D).

### The Protective Effects of OCN on 6-OHDA-Induced PC12 Cell Injury

**Figure 6:**
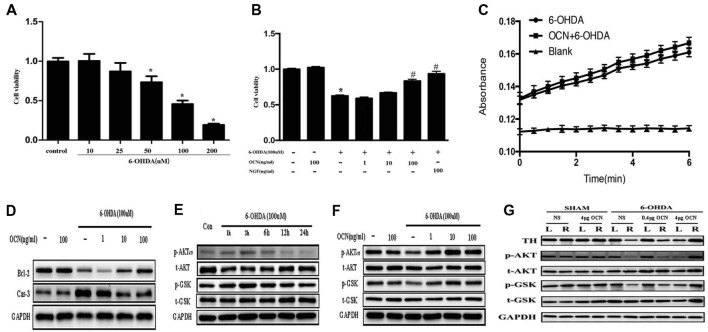
Neuroprotective effects of OCN on 6-OHDA-induced neurotoxicity. PC12 cells were incubated with different concentrations of 6-OHDA for 24 h, and cell viability was measured by cell counting kit-8 (CCK-8; **A**). Pretreatment of PC12 cells with various concentrations of OCN (1–100 ng/ml) was performed 2 h before exposure to 100 μM 6-OHDA, and the cell viability was measured at 24 h **(B)**. The effects of OCN on the auto-oxidation of 6-OHDA were measured spectrophotometrically at 490 nm using a microplate reader **(C)**. The results from western blots showed that treatment with 100 μM 6-OHDA increased the expression of caspase-3 and decreased the expression of bcl-2, and both changes were reversed by pre-incubation with OCN **(D)**. 6-OHDA decreased the expression of phosphorylated AKT (Ser473) and phosphorylated GSK3β (Ser9) at 24 h **(E)**, and both reductions were reversed by pretreatment with OCN **(F)**. Similarly, 6-OHDA injection into the right striatum also resulted in decreased expression of p-AKT and p-GSK3β, and both reductions were inhibited by direct injection of OCN into the striatum **(G)**. Values were obtained from three independent experiments. *P < 0.05 (compared with the control group), ^#^*P* < 0.05 (compared with the 6-OHDA group).

To investigate how OCN protects against neuronal degeneration, PC12 cell culture was used to identify the molecular mechanism involved in the beneficial function of OCN. 6-OHDA exposure significantly decreased the viability of PC12 cells in a dose-dependent manner after 24 h, as indicated by a CCK-8 assay (*P* < 0.001, Figure 6A). As 100 μM 6-OHDA caused 50% toxicity, we used this concentration in all following experiments. Pre-incubation of PC12 cells with OCN for 2 h significantly reversed the deteriorating impacts of 6-OHDA on cell viability (*P* < 0.001, Figure 6B). In addition, the morphology changes of the PC12 cells were also examined. With the treatment with 6-OHDA for 24 h, PC12 cells lost cellular processes, became round and detached from the bottom of the culture dish, while pretreatment with OCN dramatically ameliorated the morphological injuries imposed by 6-OHDA in PC12 cells (Supplementary Figure S3). We further evaluated the direct effect of OCN on 6-OHDA auto-oxidation, and found no effect of OCN on 6-OHDA auto-oxidation in a spectrophotometric assay (Figure 6C).

### OCN Ameliorates PC12 Cell Death Induced by 6-OHDA Through the AKT/GSK3β Signaling Pathway

We further assessed the anti-apoptotic pathway of OCN in 6-OHDA-induced cell death. OCN significantly reversed the upregulation of caspase-3 (*P* < 0.001) and decline in bcl-2 (*P* < 0.001) induced by 6-OHDA (Figure 6D). AKT-GSK3β signal transduction dysfunction has been observed in both *in vitro* and *in vivo* studies of PD patients and models (Morissette et al., 2010). Since OCN can activate the cellular survival signaling molecule AKT (Liu et al., 2017), we thus explored whether OCN can reverse dopaminergic neuronal loss in PD models through the AKT/GSK3β signaling pathway. As shown in Figure 6E, the phosphorylation levels of AKT (Ser473) and GSK3β (Ser9) were significantly decreased at 24 h after 6-OHDA incubation, although there was a transient increase within 3 h, which was consistent with previous findings (Xu et al., 2013; Guo et al., 2017). Pretreatment with OCN markedly reversed the dephosphorylation of AKT (*P* < 0.001) and GSK3β (*P* < 0.001) induced by 6-OHDA at 24 h (Figure 6F).

In addition, we verified OCN had same effect on AKT and GSK3β *in vivo*. To observe the acute change in AKT and GSK3β signaling after OCN intervention, OCN was injected intraperitoneally 2 days before the 6-OHDA injection and lasted for 2 days. Then, the striatum tissue was extracted for western blot analysis. The results demonstrated that 6-OHDA injection decreased phosphorylation of both AKT and GSK3β in the right striatum, which was inhibited by OCN i.p. administration (*P* < 0.001, Figure 6G).

**Figure 7:**
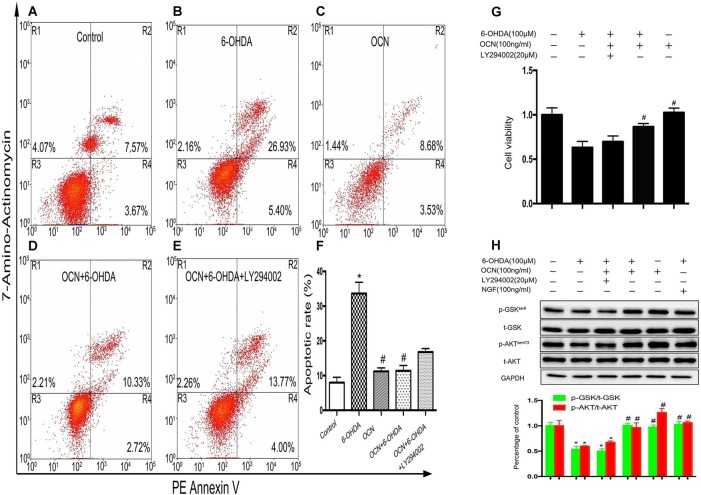
OCN protects PC12 cells from 6-OHDA-induced neurotoxicity. Apoptotic cells were measured by flow cytometry **(A–E)**. The number of apoptotic cells was counted in the R2 (AV^+^/7-amino-actinomycin, 7-AAD^+^, the late phase apoptotic cells) and R4 (AV^+^/7-AAD^−^, the early phase apoptotic cells) regions, and the apoptotic rate was calculated **(F)**. The CCK-8 test showed that OCN could attenuate the neuronal toxicity induced by 6-OHDA through activating AKT/GSK3β, while LY294002 reversed the neuroprotective effect of OCN on PC12 cells **(G)** through inhibiting the phosphorylation of AKT and GSK3β **(H)**. Values were obtained from three independent experiments. *P < 0.05 (compared with the control group), ^#^*P* < 0.05 (compared with the 6-OHDA group).

Phosphoinositide 3-kinase (PI3K) is a vital kinase involved in AKT activation (Hennessy et al., 2005). To further verify the effect of AKT/GSK3β signaling on OCN function, we assessed the neuronal protective function of OCN in the absence or presence of PI3K inhibitor LY294002. In the apoptosis analysis by flow cytometry, after PC12 cells were treated with 6-OHDA for 24 h, the percentage of apoptotic cells (in the R2 and R4 regions) increased significantly compared to that of the control (Figures 7A,B). Pretreatment with OCN significantly ameliorated the neurotoxicity induced by 6-OHDA (Figures 7C,D), suggesting a protective role of OCN inPC12 cells. However, the protective effect of OCN was attenuated in the presence of LY294002 (Figure 7E). The apoptotic rate in each group is summarized in Figure 7F (*P* < 0.001). The CCK-8 data showed that LY294002 inhibited the beneficial effect of OCN on PC12 cell viability (*P* < 0.001, Figure 7G). The levels of p-AKT were measured through western blot analysis. As shown in Figure 7H, 6-OHDA dramatically decreased the levels of p-AKT, and OCN intervention could significantly reverse its effect. However, after pretreatment with LY294002, the effect of OCN on the phosphorylation of AKT was significantly abolished (*P* < 0.001, Figure 7H).

## Discussion

The current study provided evidence that bone-derived OCN has both central and peripheral effects to reduce dopaminergic neuronal injury and improve movement deficit and limb asymmetry in a PD rat model and that the proliferation of microglia and astrocytes in the SN in PD were also dramatically inhibited by OCN interventions. These protective functions of OCN on SN dopaminergic neurons was mediated via AKT/GSK3β signaling.

Bone is now regarded as an endocrine organ modulating whole-body energy metabolism through secreting several osteokines; OCN is the most important one among them (Mera et al., 2017), and its role in brain was recently identified and thus opened a new field in this area (Oury et al., 2013; Obri et al., 2018). However, previous work mainly focused on the role of OCN in improving age-related cognitive decline and preventing anxiety and depression (Obri et al., 2018), and its neuroprotective function in neurodegenerative disorders such as PD has not been tested.

Dopaminergic neuronal degeneration in the SN is the key pathogenic feature in PD (Fearnley and Lees, 1991; Dodson et al., 2016). It was reported that in the midbrain, OCN is needed for the synthesis of monoamine neurotransmitters, as evidenced by a 20%–50% decrease in serotonin, DA and noradrenaline levels and a 15%–50% decrease in expression of genes necessary for the synthesis of these neurotransmitters in the midbrain and brainstem of OCN^−/–^ mice compared with WT mice (Oury et al., 2013). In the present study, we found that OCN levels in the CSF of PD rat model were significantly lower than in that in control rats; although we could not prove the causal effect, this novel finding indicated that lack of OCN within the brain is at least related to PD development, and its treatment might be helpful to correct the functional behavioral outcomes and pathophysiological changes in PD.

To test this hypothesis, we injected OCN intraperitoneally and into the striatum, separately. The results were encouraging: no matter whether OCN was given peripherally or centrally, it alleviated motor dysfunction in PD rat models as evaluated by OFT and cylinder tests. However, in the EBST, we failed to observe a marked decline in contralateral swings (left) compared to ipsilateral swings (right) in the 6-OHDA-lesioned rats. The results from the EBST in other reports were also conflicting; some reported an increased number of contralateral turns in 6-OHDA models compared to controls (Im et al., 2016; Ray et al., 2016), while others documented the opposite results (Henderson et al., 2003). The potential reason for these inconsistent results may be the differences in the dose and location of the 6-OHDA injection and the timing when EBST was performed, which can influence the sensitivity of EBST in evaluating motor asymmetry in unilateral 6-OHDA-lesioned rats (Roghani et al., 2002).

In addition to improvement in locomotor dysfunction, the TH-positive neuron loss in 6-OHDA-lesioned rats was also restored by OCN. Since the main pathological feature of PD is dopaminergic neuron depletion (Dodson et al., 2016), this finding adds credence to explain why OCN could ameliorate movement disorder in PD. In short, our data clearly demonstrated that either administered centrally or in a more convenient way, peripherally, OCN protects against the motor deficit and TH-positive neuron loss in PD.

To further scrutinize OCN’s neuroprotective effects at the cellular level in PD, we focused on astrocytes and microglia, since they are the most common cells in the brain and are involved in the development and progression of neurodegenerative diseases, including PD (Liddelow et al., 2017). Astrocytes are the main cells participating in damage repair after neuronal injury (Booth et al., 2017). We found that the number of astrocytes in the SN was dramatically increased in PD rats, while OCN could lessen this pathological alteration, indicating less dopaminergic neuronal loss in OCN-treated PD rats. It was also noticed that in PD rat models, the proliferation of astrocytes was especially obvious in the SN but not in the striatum. The potential reason for this phenomenon might be a result of the more severe neuronal injury in the SN, as the dopaminergic neuronal cellular bodies are located in the SN, not the striatum (Geffen et al., 1976). In addition, overactivated microglia-induced chronic inflammation is essential for the pathogenesis of PD (Tansey and Goldberg, 2010; Ros-Bernal et al., 2011; Dzamko et al., 2015). Our results indicated that OCN could partially reduce the amount of microglia in the SN and striatum and inhibit the expression of IL-1 and TNF-α in the striatum of PD rats. These observations may have clinical implications, since there were reports that suppressing the activation and proliferation of microglia can reverse dopaminergic neuron dysfunction and improve PD symptoms (Joers et al., 2017).

We next explored the molecular mechanism responsible for the neuroprotective effects of OCN. Defective AKT/GSK3β signaling is a crucial pathophysiological feature in the development of PD (Szego et al., 2017). Indeed, 6-OHDA decreases the phosphorylation of AKT at Ser473 and GSK3β at Ser9 (Tiong et al., 2010; Singh et al., 2016), and our *in vitro* experiments revealed that OCN protected PC12 from the 6-OHDA-induced cell toxicity and apoptosis via AKT/GSK3β signaling. Actually, the capability of OCN to activate the AKT-GSK3β pathway was described in C2C12 myoblasts (Liu et al., 2017). In addition, our apoptotic analysis revealed that the neuroprotection afforded by OCN in PC12 cells was dramatically abolished after the inhibition of AKT and GSK3β phosphorylation. These data indicated that OCN reduces dopaminergic neuronal injury induced by 6-OHDA via the AKT/GSK3β signaling pathway.

There were some other questions and limitations related to this study worthy of discussion. Since OCN improves muscle power (Mera et al., 2016b) and male fertility in mice (Oury et al., 2011), it could be argued that OCN’s protective role in PD rats might be mediated through these peripheral effects. Although this possibility could not be excluded, it is unlikely based on the following reasons: (1) previous mouse studies have confirmed that OCN’s antianxiety and learning and memory-favoring effects were independent of its metabolic functions (Oury et al., 2013); (2) the OCN concentration in the CSF was much lower in the PD rat model, while its level in the peripheral blood was comparable with that in the control rats, suggesting the involvement of central OCN in PD development is more obvious; and (3) the OCN receptor GPR158 was indeed present in rat brain as revealed in this study.

Another intriguing question elicited by the current study is how OCN administered in the striatum could provide neuroprotective impact in a disease with the main pathological abnormality in the SN. At this time, it is hard to answer due to a lack of data from the current study and the literature. However, looking at the way the PD model is established may provide some clues to address this question. Injection of 6-OHDA into the striatum is one of classic ways to generate the PD model (Lindholm et al., 2007), and it achieves a retrograde disappearance of nigrostriatal DA neurons by a “dying back” mechanism (Blandini et al., 2008). This retrograde degeneration induced by 6-OHDA, which is regarded as a kind of chemical axotomy that effectively isolates the nigral DA neurons from their target structure, has been proposed to result from a loss of striatum-derived trophic support (Sauer and Oertel, 1994). Actually, the striatum is the major target of midbrain DA projections, and loss of neural activity in the striatum is also responsible for the movement impairment in PD (Mazzoni et al., 2007; Panigrahi et al., 2015). Thus, the striatum and SN are not only anatomically and physiologically connected, their pathological alterations in PD are also tightly interwoven. Therefore, the findings that OCN administered in the striatum could exert effects on the SN are reliable.

In addition, it was recently reported that the βγ subunit of the heterotrimeric G proteins interacts with the DA transporter (DAT) and modulates brain DA homeostasis by promoting DA efflux (Garcia-Olivares et al., 2017). Since GPR158 is a G protein-coupled receptor, and our study confirmed the wide presence of GPR158 in the rat brain, including the SN and striatum, and the binding of OCN and GPR158 in the striatum, it could thus be speculated that activation of GPR158 by OCN in the striatum might lead to functional alterations in the DAT and DA neurons. However, it must be acknowledged that in our *in vitro* experiments, OCN’s effects on PC12 cells were irrelevant of DAT. Thus, a great deal of work is warranted to elucidate the detailed mechanisms explaining why OCN infused in the striatum could reduce DA neuronal loss in the SN of PD rats. In this study, we found the OCN level in the CSF was significantly decreased as compare to control, but the reason was unknown. In fact, cystatin C and Nurr1, which could regulate the bone metabolism and OCN expression (Pirih et al., 2004; Danjo et al., 2007), were tightly associated with the development of PD (Wei et al., 2016; Zou et al., 2017; Weng et al., 2018). However, whether OCN was involved in the process need to be further explored.

There are several limitations in the current study. Since this study focus on the role of OCN on striatal function, no OCN alone treated group was set up in the OCN i.p. injection experiments. However, the results from OCN alone injection into striatum showed no obvious effect on rat’s brain function, suggesting OCN i.p. injection alone might not influence brain function. PI3K inhibitor LY294002 was not administered centrally or systemically to test whether it could abolish the beneficial efficacy of OCN in PD rats. In addition, a Gpr158 knockout PD model was not used in our study to identify whether OCN truly exerts its neuroprotective efficacy through this central receptor in PD rats.

In conclusion, the present study establishes that both i.p. and i.s. injection of OCN protects against dopaminergic neuronal loss and improves motor deficits in the 6-OHDA-induced PD rat model. The AKT/GSK3β signaling pathway is implicated in the neuroprotective effects of OCN. Based on the novel findings from Karsenty’s group on OCN’s regulation of cognition, our study provides another piece of evidence to demonstrate the effect of OCN on the brain. The potential use of OCN-based therapy in PD and other neurodegenerative disorders is worthy of further investigations.

## Author Contributions

XG and CS performed the experiments and wrote the manuscript. YH and GZ carried out select experiments. BT, LS along with HZ interpreted the data. GN, SL and JL conceived the experiments of this study and modified the manuscript.

## Conflict of Interest Statement

The authors declare that the research was conducted in the absence of any commercial or financial relationships that could be construed as a potential conflict of interest.
