# Supplementary material for: Osteocalcin Ameliorates Motor Dysfunction in a 6-Hydroxydopamine-Induced Parkinson’s Disease Rat Model Through AKT/GSK3β Signaling
Source: Front Mol Neurosci. 2018 Sep 27;11:343. doi: 10.3389/fnmol.2018.00343 (PMC6170617; doi:10.3389/fnmol.2018.00343)
Supplement: Supplementary file 1 [file Data_Sheet_1.docx]

***Supplementary Material***

**Osteocalcin ameliorates motor dysfunction in a 6-hydroxydopamine hydrobromide-induced Parkinson’s disease rat model through AKT/GSK3β signaling**

**Xing-zhi Guo^#^, Chang Shan^#^, Yan-fang Hou, Geng Zhu, Bei Tao, Li-hao Sun, Hong-yan Zhao, Guang Ning^*^, Sheng-tian Li^*^, Jian-min Liu^*^**

***Correspondence:** Guang Ning: [gning@sibs.ac.cn](mailto:gning@sibs.ac.cn); Sheng-tian Li: [lstian@sjtu.edu.cn](mailto:lstian@sjtu.edu.cn); Jian-min Liu:

[ljm10586@rjh.com.cn](mailto:ljm10586@rjh.com.cn).

**#:** These two authors contributed equally to this work

**Supplementary Figure 1.** The experiment schedule for OCN intervention on 6-OHDA induced PD rat models.

**Supplementary Figure 2.** Injection of 6-OHDA into ST increased the expression of TNF-α and IL-1β in the ST, and OCN given systemically had a trendecncy to reduce the expression of TNF-α and IL-1β 6-OHDA induced PD rat models.

**Supplementary Figure 3.** The effect of OCN intervention on the morphorlogy changes of the PC12 cells after treated with 6-OHDA. With the treatment with 6-OHDA for 24h, PC12 cells lost cellular processes, became round and detached from the bottom of the culture dish, while pretreatment with OCN dramatically ameriotrated the morphological injuries imposed by 6-OHDA in PC12 cells.

**Supplementary Figure 1.**

**
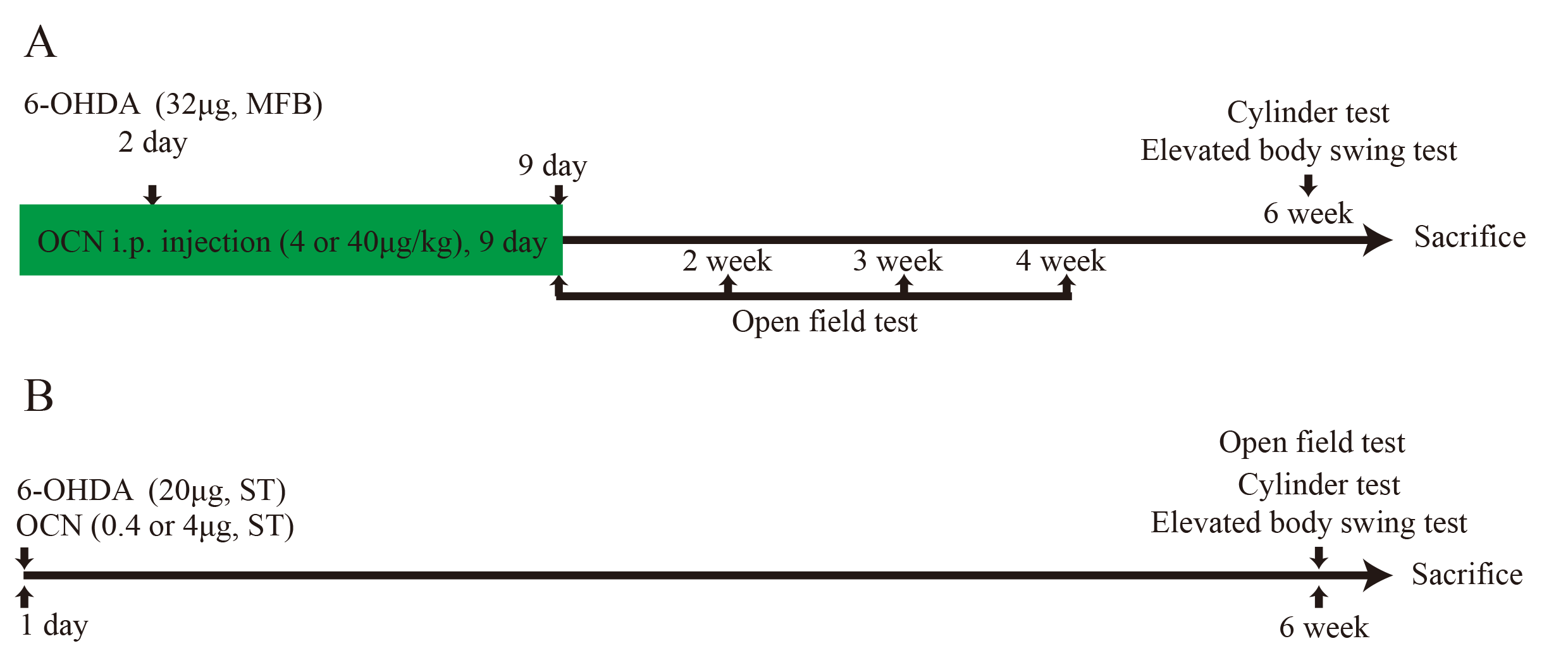
**

**Supplementary Figure 2.**

**
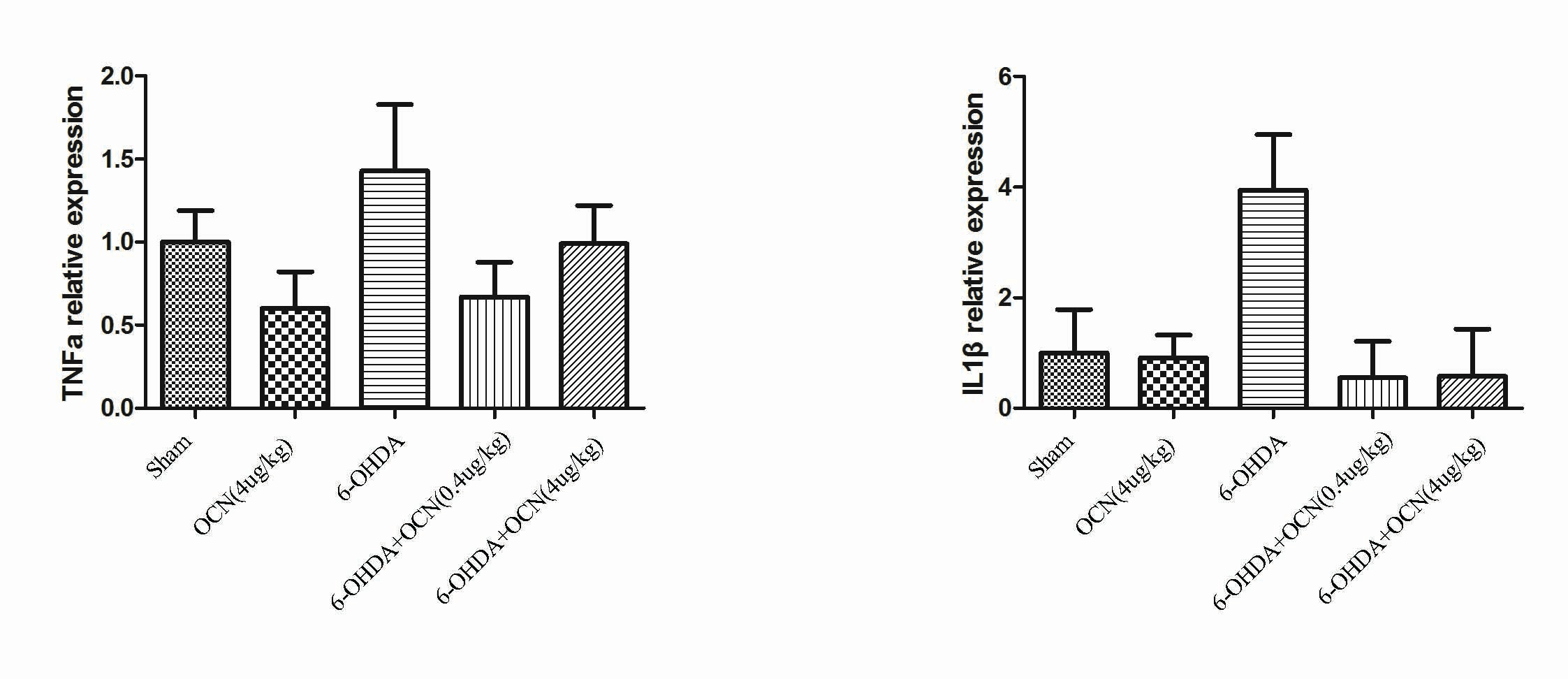
**

**Supplementary Figure 3.**

**
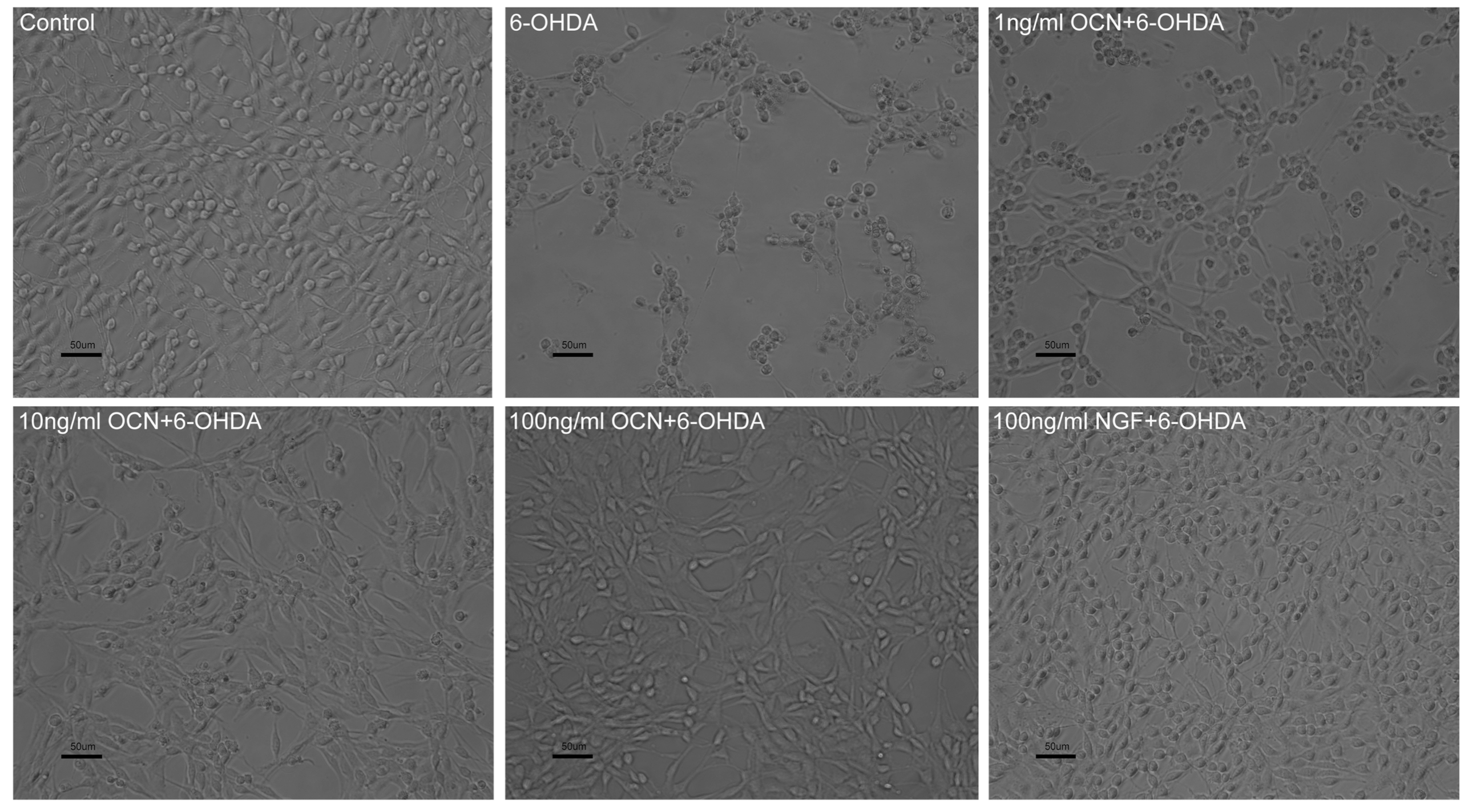
**

| **Supplementary Table 1** Results of open field, cylinder test and elevated body swing test in SD rats (mean ± SEM) of intraperatoneal-injection OCN. | | | | |
| --- | --- | --- | --- | --- |
| Tests | Sham (n=10) | 6-OHDA (n=8) | 6-OHDA+4μg/kg OCN (n=9) | 6-OHDA+40μg/kg OCN (n=9) |
| Open field test |  |  |  |  |
| Total distance (m) |  |  |  |  |
| 1st week | 12.32 ± 1.58 | 14.57 ± 1.79 | 12.73 ±0.72 | 9.38 ±1.70 |
| 2nd week | 11.28 ± 0.85 | 9.42 ± 1.37δ | 12.43 ±1.21 | 10.42 ±0.76 |
| 3rd week | 12.91 ± 0.84 | 8.59 ± 0.88#δδ | 12.36 ±0.98 | 10.41 ±1.48 |
| 4th week | 10.36 ± 1.36 | 4.71 ± 0.97##δδ | 10.37±1.01** | 8.67 ±1.16 |
| Rearing (number) |  |  |  |  |
| 1st week | 25.00 ±4.25 | 23.13 ± 7.73 | 22.89 ±2.32 | 17.11 ±1.79 |
| 2nd week | 20.2 ± 2.73 | 17.86 ± 2.26 | 19.78 ±2.76 | 23.11 ±2.73 |
| 3rd week | 26.1 ± 2.15 | 13.63 ± 2.57# | 21.78 ±1.78 | 24.78 ±4.49* |
| 4th week | 22.22 ± 4.02 | 10.00 ± 1.94δ | 19.44±3.63 | 18.78 ±4.43 |
| Cylinder test (left/right) 4th week | 0.997 ± 0.015 | 0.585 ± 0.062## | 0.835 ± 0.031* | 0.756± 0.088## |
| Elevated body swing test (left/right) 4th week | 0.797 ± 0.077 | 3.031 ± 0.649# | 2.344 ± 0.603# | 1.770 ± 0.223 |

Note: * significantly compare to the 6-OHDA group. # significantly compare to the Sham group. δ significantly compare to 1st week within group. *<0.05, **<0.001. #<0.05, ##<0.001, δ<0.05, δδ<0.001.

**Supplementary Table 2** Results of open field, cylinder test and elevated body swing test in SD rats (mean ± SEM) of ST-injection OCN.

| Tests | Sham (n=11) | Sham+4μg OCN (n=10) | 6-OHDA (n=9) | 6-OHDA+0.4μg OCN (n=10) | 6-OHDA+4μg OCN (n=9) | 6-OHDA+1μg NGF (n=11) |
| --- | --- | --- | --- | --- | --- | --- |
| Open field test |  |  |  |  |  |  |
| Total distance (m) | 31.92 ± 3.08 | 27.60 ± 4.08* | 13.53 ± 2.67## | 25.42 ±3.41 | 27.80 ±3.88* | 27.43 ±2.49* |
| Number of rearing (times) | 24.00 ± 2.72 | 26.80 ±2.59** | 13.11 ±1.78# | 19.10 ± 3.91 | 16.78 ± 2.68 | 15.91 ± 2.34 |
| Cylinder test (left/right) | 0.980 ± 0.049 | 1.093 ± 0.052* | 0.690 ± 0.121 | 0.755 ± 0.096 | 0.864 ± 0.089 | 0.992 ± 0.086 |
| Elevated body swing test (left/right) | 1.048 ± 0.169 | 1.135 ± 0.205 | 0.709 ± 0.160 | 1.076 ± 0.122 | 0.733 ± 0.103 | 1.010 ± 0.164 |
| Note: * significantly compare to the 6-OHDA group. #significantly compare to the Sham group. *<0.05, **<0.001. #<0.05, ##<0.001. | | | | |  |  |
